# MiR-30a Inhibits the Epithelial—Mesenchymal Transition of Podocytes through Downregulation of NFATc3

**DOI:** 10.3390/ijms161024032

**Published:** 2015-10-12

**Authors:** Rui Peng, Li Zhou, Yuru Zhou, Ya Zhao, Qianyin Li, Dongsheng Ni, Yanxia Hu, Yaoshui Long, Jianing Liu, Zhongshi Lyu, Zhaomin Mao, Yue Yuan, Liyuan Huang, Hui Zhao, Ge Li, Qin Zhou

**Affiliations:** 1 The Divsion of Molecular Nephrology and the Creative Training Center for Undergraduates, the M.O.E. Key Laboratory of Laboratory Medical Diagnostics, the College of Laboratory Medicine, Chongqing Medical University, Chongqing 400016, China; E-Mails: pengrui911@foxmail.com (R.P.); shmily900519@foxmail.com (L.Z.); zhouyuru93@sina.com (Y.Z.); lqianyin@gmail.com (Q.L.); dongshengni@outlook.com (D.N.); hyx_zuichu@outlook.com (Y.H.); lys960110@sina.com (Y.L.); keithljn@sina.cn (J.L.); zhongshilyu@sina.com (Z.L.); zhaominmao@sina.com (Z.M.); yyokyy1126@hotmail.com (Y.Y.); lyhuang0603@sina.com (L.H.); 2 The College of Laboratory Medicine, Chongqing Medical University, Chongqing 400016, China; 3 The First Hospital of Xi’an, Xi’an 710002, China; E-Mail: zhaoya2508@sina.com; 4 Key Laboratory for Regenerative Medicine, Ministry of Education, School of Biomedical Sciences, Faculty of Medicine, The Chinese University of Hong Kong, Hong Kong, China; E-Mail: zhaohui@cuhk.edu.hk; 5 Core Facility of Experiments Training, Chongqing Medical University, Chongqing 400016, China; E-Mail: geli@cqmu.edu.cn

**Keywords:** microRNA-30a (miR-30a), podocyte, epithelial-to-mesenchymal transition (EMT), nuclear factor of activated T cells 3 (NFATc3)

## Abstract

MicroRNAs (miRNAs) possess an important regulating effect among numerous renal diseases, while their functions in the process of epithelial-to-mesenchymal transition (EMT) after podocyte injury remain unclear. The purpose of our study is to identify the potential functions of miR-30a in EMT of podocytes and explore the underlying mechanisms of miR-30a in the impaired podocytes. The results revealed that downregulation of miR-30a in podocyte injury animal models and patients, highly induced the mesenchymal markers of EMT including Collagen I, Fibronectin and Snail. Furthermore, overexpression of miR-30a enhances epithelial markers (E-cadherin) but diminished mesenchymal markers (Collagen I, Fibronectin and Snail) in podocytes. In addition, we established miR-30a target NFATc3, an important transcription factor of Non-canonical Wnt signaling pathway. More importantly, our findings demonstrated that the augmentation of miR-30a level in podocytes inhibits the nuclear translocation of NFATc3 to protect cytoskeleton disorder or rearrangement. In summary, we uncovered the protective function of miR30a targeting NFATc3 in the regulation of podocyte injury response to EMT.

## 1. Introduction

Podocyte depletion, one of the common phenomena in chronic kidney diseases (CKDs) such as focal segmental glomerulosclerosis (FSGS), diabetic nephropathy (DN), membranous glomerulopathy, and lupus nephritis, may be attributed to the defects of the podocytes, glomerular basement membrane (GBM), endothelial cells, and/or negatively charged proteins present on the three layers [1]. Podocytes are formed by interdigitating cellular extensions, which are bridged by slit diaphragms, which is similar to an adherence-like intercellular junction. Slit diaphragms of podocytes select the size of proteins that freely infiltrate the filtration barrier, exclusive water and small solutes. Recent studies have found that many molecular structures of the slit diaphragm have functional work in its integrity [2,3,4], such as zonula occludens-1 (ZO-1), nephrin, and P-cadherin.

The injury of podocytes would destroy the structure and function of the slit diaphragms, and might undergo epithelial-to-mesenchymal transition (EMT) [5], a reversal of embryogenesis presented to tubulointerstitial fibrosis and diabetic kidney. EMT induces three primary changes in cellular phenotype [6,7]. Morphology of cells converts from cobblestone-like epithelial cells to spindle-shaped mesenchymal cells. Alteration of functions connects with the changes of settled cells to migratory cells. Marker gene expression varies, including loss of ZO-1 and E-cadherin as markers of epithelial cells, and upregulation of Collagen I and Fibronectin as markers for mesenchymal cells. Furthermore, markers of proteins switch from cytokeratin to vimentin. Moreover, EMT is considered to contribute to renal fibrogenesis and defined as a source of the phenotype and functional properties of transformation of epithelial cells into mesenchymal fibroblasts. Transforming growth factor beta (TGFβ) is an essential factor for EMT and fibrosis of tubular epithelial cells in Diabetic kidney disease [8]. More interestingly, nuclear factor of activated T cells (NFAT) also plays an important role in EMT. NFATc2/c3/c4 activates EMT in the myocardium of embryonic mouse by repressing VEGF expression [9]. Moreover, NFAT inducing activation of the fibronectin promoter and upregulation of fibronectin in podocytes, dependent on the calcium/calmodulin pathway and Rho kinase, finally, leads to podocyte injury and proteinuria [10]. These results strongly support that EMT is a key step in embryogenesis and chronic kidney diseases, especially the podocytes in response to various injuries and diseases. However, increased efforts must be made to understand the latent mechanisms of EMT in podocyte injury.

MicroRNAs (miRNAs) are a large family of endogenous, single stranded, small non-coding RNAs with 18-25 nucleotides in length, which downregulate target gene expression by binding to the 3ʹ-untranslated region (3ʹUTR) of target mRNAs leading to mRNA degradation or translational inhibition. Multiple studies have demonstrated that miRNAs regulate most biological processes of cells, including proliferation, differentiation, senescence and apoptosis, and that severe diseases can be attributed to abnormal expression of miRNA. Recent studies indicated miRNAs can affect various gene expression in kidney diseases. For instance, miR-195 could facilitate mouse podocytes apoptosis by targeting BCL2 under high-glucose conditions [11]. Another example is miR-93 that modulates the pathogenesis of diabetic nephropathy through repression of VEGF expression and its downstream signaling [12]. Meanwhile, accumulating studies have demonstrated that miRNAs are also linked to EMT through dysregulation of EMT-related genes. MiR-34a, decreased in hypoxia-induced renal tubular epithelial cells, could promote EMT by directly targeting Notch1 and Jagged1 [13]. Another study showed miRNA-200b causes EMT in kidney proximal tubular cells by targeting TGF-β1 [14]. All these studies demonstrate that miRNAs play indispensable roles in the process of glomerular diseases, especially the EMT-associated disharmonies. However, the underlying mechanisms of miRNAs on EMT remain largely unknown.

MiR-30a is specifically expressed in collecting duct cells and podocytes [15] and is identified as a biomarker in the urine of FSGS patients [16]. In addition, miR-30a was reportedly associated with EMT in peritoneal fibrosis [17] and several cancers, including non-small cell lung cancer (NSCLC) [18,19], gastric cancer [20] and hepatocellular carcinoma (HCC) [21]. Despite these findings, the molecular mechanisms of miR-30a in the EMT of podocyte injury need further investigation. A large number of targets for miR-30a were predicted using bioinformatic analyses including: miRwalk (http://www.umm.uni-heidelberg.de/apps/zmf/mirwalk/), miRanda (http://microrna.org), and Target Scan release 6.2 (http://www.targetscan.org). Among these targets, NFATc3 has been revealed to be involved in EMT as previously described [9]. In our study, we examined the role and potential mechanism of miR-30a in podocyte EMT. We found that miR-30a was repressed in patients with FSGS and a mouse model of podocyte injury. It is also downregulated in cultured podocytes that have been treated with Adriamycin (ADR) [22]. We further demonstrated that ectopic expression miR-30a inhibits EMT of podocytes by targeting NFATc3, and downregulation of miR-30a results in promotion of EMT. Thus, we identified miR-30a as having a novel role in the process of podocyte EMT, which may provide a new understanding of molecular mechanisms about podocyte injury and provide a new potential target for the treatment of podocytopathy.

## 2. Results

### 2.1. miR-30a Is Downregulated in Podocyte Injury

**Figure 1:**
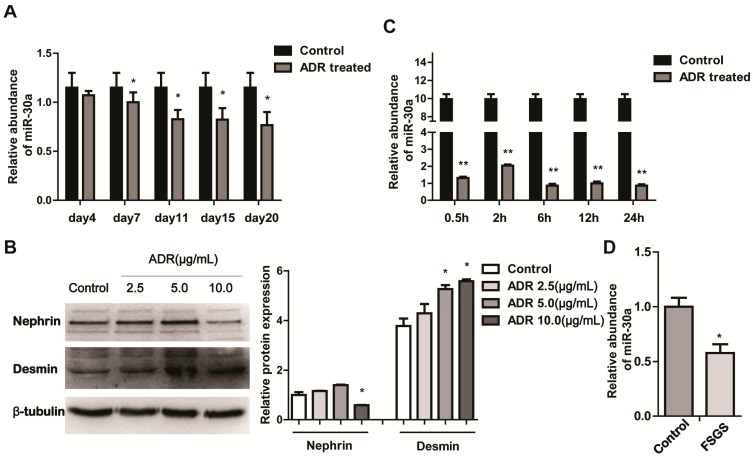
MiR-30a is Downregulated in Podocyte Injury. (**A**) RT-qPCR analysis of miR-30a expression levels in glomeruli from BALB/c mouse after Adriamycin (ADR; 10.5 mg/kg) injection for 4, 7, 11, 15 and 20 days (*n* = 3, mean ± SD); (**B**) Western blot analysis of Nephrin and Desmin expression levels in cultured MPC5 cells treated with ADR at different doses (2.5, 5.0 and 10.0 µg/mL); Quantitative analysis of Nephrin and Desmin protein levels after normalization with β-tubulin; (**C**) RT-qPCR analysis of miR-30a expression levels in cultured MPC5 cells treated with ADR (5.0 µg/mL) at different time points (0.5, 2, 6, 12 and 24 h); (**D**) RT-qPCR analysis of miR-30a expression levels in the glomeruli from one patient with focal segmental glomerulosclerosis (FSGS) and one patient with kidney rupture (Control). The relative amount of miR-30a was normalized to U6. All data are presented as means ± SD from three independent experiments. * *p* < 0.05, ** *p* < 0.01, indicate statistically significant *and* highly statistically significant differences, respectively.

To investigate whether miR-30a is involved in the pathogenesis of podocyte injury, we first examined miR-30a expression in the glomeruli isolated from mice intravenously injected with Adriamycin (ADR, 10.5 mg/kg). This model was widely used for podocyte injury [22,23]. We found that miR-30a decreased after intravenous injection of ADR in a time-dependent manner in all five groups compared with the control group (Figure 1A), and the effect peaked at 11 days post ADR treatment. We further examined the expression of miR-30a in ADR-induced podocyte injury *in vitro*, and measured the podocyte injury induced in dose gradients by western-blot (Figure 1B). The expression of proteins related to podocyte injury was detected by western-blot. Nephrin, a podocyte marker, was significantly reduced (10 µg/mL ADR treatment), but as for desmin, a podocyte injury marker [24], was observably increased (5 µg/mL ADR treatment). Meanwhile, miR-30a was significantly downregulated in a time-dependent manner (Figure 1C; 5 µg/mL ADR treatment). We next confirmed clinical relevance of miR-30a in glomeruli isolated from one patient with FSGS identified by renal biopsy [25] and another patient with kidney rupture due to violence (Control). As expected, the expression of miR-30a was lower in the glomeruli from the FSGS patient compared to those of the control (Figure 1D), suggesting that miR-30a is downregulated during podocyte injury.

**Figure 2:**
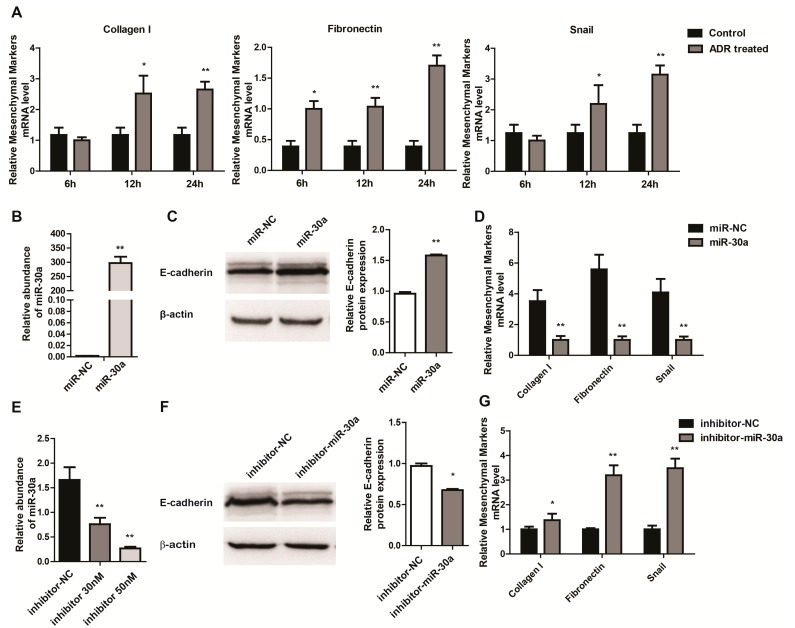
MiR-30a Inhibits Epithelial-to-Mesenchymal Transition in Podocytes. (**A**) RT-qPCR analysis of mesenchymal markers (Collagen I, Fibronectin and Snail) expression levels in cultured MPC5 cells treated with ADR (5.0 µg/mL) at different time points (6, 12 and 24 h); (**B**) RT-qPCR analysis of miR-30a expression levels in cultured MPC5 cells transfected with miR-30a (30 nM) or miR-NC; (**C**) Western blot analysis of epithelial markers (E-cadherin) expression levels in cultured MPC5 cells transfected with miR-30a (30 nM) or miR-NC. Quantitative analysis of E-cadherin protein levels after normalization with β-actin; (**D**) RT-qPCR analysis of mesenchymal markers (Collagen I, Fibronectin and Snail) expression levels in cultured MPC5 cells transfected with miR-30a (30 nM) or miR-NC; (**E**) RT-qPCR analysis of miR-30a expression levels in cultured MPC5 cells transfected with inhibitor-30a (30 or 50 nM) or inhibitor-NC; (**F**) Western blot analysis of epithelial markers (E-cadherin) expression levels in cultured MPC5 cells transfected with inhibitor-30a (50 nM) or inhibitor-NC. Quantitative analysis of E-cadherin protein levels after normalization with β-actin; (**G**) RT-qPCR analysis of mesenchymal markers (Collagen I, Fibronectin and Snail) expression levels in cultured MPC5 cells transfected with inhibitor-30a (50 nM) or inhibitor-NC. The relative amount of miR-30a and mRNA was normalized to that of control RNA (U6) and 18s, respectively. All data are presented as means ± SD from three independent experiments. * *p* < 0.05, ** *p* < 0.01, indicate statistically significant and highly statistically significant differences, respectively.

### 2.2. miR-30a Inhibits Epithelial-to-Mesenchymal Transition in Podocytes

As previously mentioned, we speculated that miR-30a may serve as a key factor in the pathogenesis of EMT in podocyte injury. To test this hypothesis, we examined the expression levels of mesenchymal markers, Collagen I, Fibronectin and Snail, in time-dependent manners after ADR treatment. In contrast to miR-30a, which was downregulated rapidly from 0.5 h after stimulation (Figure 1B), the mesenchymal markers increased slowly and reached its peak at least 24 h after ADR treatment (Figure 2A), which suggested a possible regulatory role of miR-30a in EMT by podocyte injury. To investigate whether the alteration of miR30a could affect the EMT of podocytes, we transfected miR-30a into MPC5 cells. The real time PCR analysis indicated that miR-30a was successfully expressed in MPC5 cells after transfection (Figure 2B). And with the overexpression of miR-30a in podocytes, the expression of epithelial marker, such as E-cadherin, were significantly increased, as confirmed by western-blot (Figure 2C). While mesenchymal markers, such as Collagen I, Fibronectin and Snail, were repressed by miR-30a, which were determined by real time PCR (Figure 2D). To further determine how miR-30a regulated EMT of podocytes, we silenced miR-30a expression in the MPC5 cells by transfecting miR-30a inhibitor (Figure 2E). miR-30a silenced MPC5 cells had lower expression of epithelial markers (Figure 2F) and higher expression of mesenchymal markers (Figure 2G) compared to negative control, which indicated miR-30a function for inhibiting EMT in podocytes. Taken together, these data indicated that miR-30a inhibits EMT in podocytes.

### 2.3. miR-30a Targets the 3ʹUTR of NFATc3 and Inhibits the Expression of NFATc3

**Figure 3:**
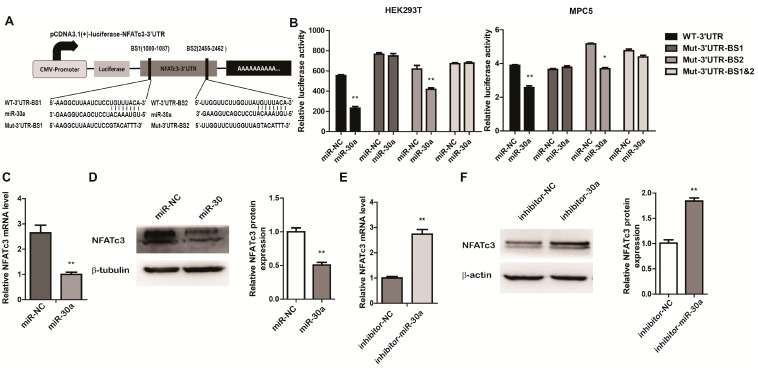
MiR-30a Targets the 3ʹUTR of NFATc3 and Inhibit the Expression of NFATc3. (**A**) Schematic presentation of two binding sites of miR-30a in the 3ʹUTR of NFATc3 and NFATc3 3ʹUTR reporter constructs, including wild type and mutant vectors; (**B**) Dual-luciferase reporter assays demonstrate that the wild-type and mutation-BS2 reporter of NFATc3 3ʹUTR suppressed activity in HEK293T cells and MPC5 cells transiently transfected with miR-30a, but not in mutation-BS1 and mutation-BS 1 and 2; (**C**) RT-qPCR and (**D**) Western blot analysis of NFATc3 expression levels in cultured MPC5 cells transfected with miR-30a (30 nM) or miR-NC. Quantitative analysis of NFATc3 protein level after normalization with β-tubulin; (**E**) RT-qPCR and (**F**) Western blot analysis of NFATc3 expression levels in cultured MPC5 cells transfected with inhibitor-30a (50 nM) or inhibitor-NC. Quantitative analysis of NFATc3 protein level after normalization with β-actin. The relative amount of NFATc3 mRNA was normalized to 18 s. All data are presented as means ± SD from three independent experiments. * *p* < 0.05, ** *p* < 0.01, indicate highly statistically significant differences, compared with miR-NC or inhibitor-NC, respectively.

We next examined whether the alteration of miR-30a was linked to the concomitant regulation of its target genes in EMT of podocytes. To investigate this, we predicted NFATc3 as an MiR-30 target using the three miRNA target analyzing databases, miRwalk, TargetScan and miRanda. To confirm whether miR-30a could directly target NFATc3-3ʹ untranslated region (UTR), we cloned 3ʹUTR of NFATc3 into a luciferase reporter vector (pcDNA3.1-luciferase). The 3ʹUTR of NFATc3 includes two binding sites perfectly complementary in sequence to the seed sequence of miR-30a (Figure 3A). These reporters were transfected into HEK293T cells and MPC5 cells along with miR-30a mimics or a negative control of miRNAs mimics (miR-NC) and luciferase activity was detected by a dual-luciferase system. Luciferase activity was suppressed in the presence of the miR-30a mimic compared with the negative control (Figure 3B). The specific regulation of NFATc3 by miR-30a was confirmed by respectively mutating the seed sequence of the position 1080-1087 (BS1), 2455-2462 (BS2) of the NFATC3 3ʹUTR and both of them (BS1 and 2) (Figure 3A). Suppression of luciferase activity was not observed when the binding site of BS2 and BS1 and 2 was mutated in the 3ʹUTR of NFATc3, supporting the view that the position 1080–1087 (BS1) of the NFATC3 3ʹUTR is a directly target of miR-30a (Figure 3B). Further evidence of the impact of miR-30a on NFATc3 was measured by the expression of NFATc3 by western blot and real-time PCR. NFATc3 was significantly downregulated after transfection with miR-30a mimics (Figure 3C), but increased after transfection of the inhibitor of miR-30a (Figure 3D), which suggests that miR-30a can inhibit NFATc3 expression at the protein level. These results demonstrate that NFATc3 is a target of miR-30a, and suggest that miR30a can downregulate the expression of NFATc3 both at RNA and protein levels.

### 2.4. NFATc3 Is Upregulated in Podocyte Injury

**Figure 4:**
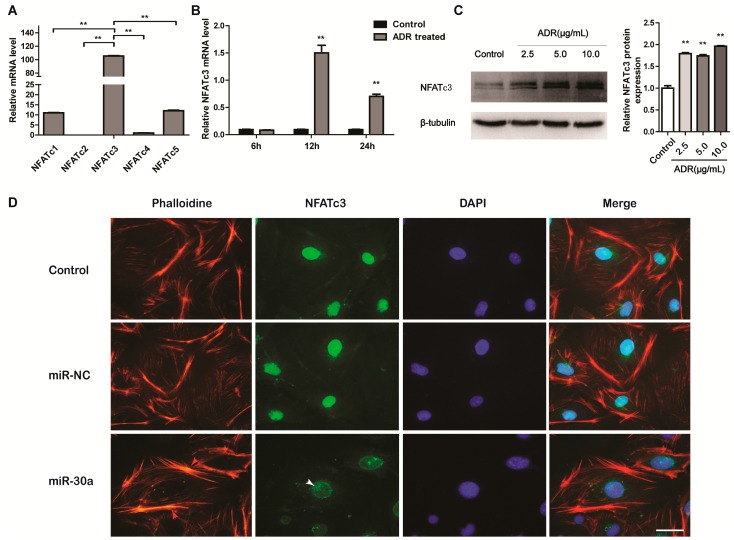
NFATc3 is Upregulated in Podocyte Injury. (**A**) NFAT family expression profile in cultured MPC5 cells was measured by RT-qPCR; (**B**) RT-qPCR analysis of NFATc3 mRNA expression levels in cultured MPC5 cells treated with ADR (5.0 µg/mL) at different time points (6, 12 and 24 h); (**C**) Western blot analysis of NFATc3 expression levels in cultured MPC5 cells treated with ADR at different doses (2.5, 5.0 and 10.0 µg/mL). Quantitative analysis of NFATc3 protein level after normalization with β-tubulin; (**D**) F-actin staining with phalloidin (red) and immunofluorescence microscopy of NFATc3 (green) in cultured MPC5 cells transfected with miR-30a (30 nM) or miR-NC, demonstrating miR-30a protects podocyte cytoskeleton disorder or rearrangement (red arrow) by inhibiting the nuclear translocation of NFATc3 (white arrow). Scale bar represents 30 µm. All data are presented as means ± SD from three independent experiments. ** *p* < 0.01, indicate highly statistically significant differences compared with no ADR-treatment (Control).

We next explored the mechanism of NFATc3 in podocyte Injury. Taking into account the differential expression of all the members of the NFAT family in podocytes, the real time PCR analysis was performed and the result demonstrated that NFATc3 was expressed more abundantly than other members (Figure 4A). Subsequently, NFATc3 has been shown to mediate podocyte injury, and its activation was observed in ADR-induced podocyte injury, in dose-dependent (Figure 4B; 24 h after ADR treatment) and time-dependent (Figure 4C; 5 µg/mL ADR treatment) manners. Activation of NFATc3 appearing in the podocyte injury suggested that NFATc3 is an important regulator in the response to podocyte injury.

As a transcription factor, when dephosphorylated by calcineurin, the location of NFAT can transfer from the cytoplasm to the nucleus [26] and induce activation of the fibronectin protein [10] in podocytes. With many different factors, podocyte cytoskeleton disorder or rearrangement could lead to the dysfunction and injury of podocytes [27]. Therefore, we wondered whether miR-30a protects podocytes by inhibiting the translocation of NFATc3 into the nucleus. When miR-30a mimics were transfected into MPC5 cells, the number of stress fibers was enhanced and located around the cytomembrane compared with miR-NC and untreated groups (Control) (Figure 4D). Importantly, the nuclear translocation in podocytes was inhibited by the miR-30a (Figure 4D). Taken together, the results indicate that miR-30a protects podocyte cytoskeleton disorder or rearrangement by inhibiting the nuclear translocation of NFATc3.

## 3. Discussion

Podocyte dysfunction, as found in hypertrophy, dedifferentiation, detachment, and apoptosis, plays a central role in numerous kidney diseases [28,29], such as FSGS and diabetic nephropathy. In this study, we have shown that miR-30a was significantly decreased in both ADR-induced glomeruli of mice and cultured podocytes, and the same result in glomeruli of FSGS patients. The fact suggests that miR-30a may be a clinically relevant biomarker for glomerulus and podocyte injury. Multiple factors could account for the podocyte injury [30,31], and the mechanisms underlying podocyte injury remain ambiguous. In our study, we focused on investigating the function of miR-30a putative target NFATc3, an important transcription factor of the non-canonical Wnt signaling pathway, in podocyte injury. The downregulation of NFATc3 is associated with the concomitant overexpression of miR-30a in podocytes, suggesting that miR-30a might be a novel beneficial factor for podocytes and protect podocytes from impairment through targeting NFATc3.

Podocyte depletion often appears in incipient-stage renal diseases accompanied with prominent proteinuria. Despite a recent study indicating that miR-30s could protect the podocyte cytoskeleton from damage and apoptosis [32], early-stage change after podocyte injury was not observed. However, EMT could be a primary reason leading to podocyte dysfunction, proteinuria, and glomerulosclerosis [33]. Our study validates EMT as a bona fide stage after podocyte injury and demonstrates that the mesenchymal markers of EMT are activated in ADR-treated podocytes. In this study, we found that the alteration of epithelial markers was upregulated in the presence of miR-30a mimics and reduced when cells are transfected with the miR-30a inhibitor. On the contrary, the mesenchymal markers of EMT in podocytes was suppressed in the presence of miR-30a mimics and enhanced when cells are transfected with miR-30a inhibitor. All these results suggest that the suppression of epithelial-mesenchymal transition in podocytes is associated with enhanced expression of miR-30a, thus demonstrating that miR-30a may be a potential mechanism to prevent EMT in podocytes. In addition, the findings of this study show that miR-30a is downregulated in ADR-treated podocytes, however the underlying mechanisms of ADR on the regulation of miR-30a in podocytes remain unclear and requires further research.

MiRNAs are a large family of short endogenous noncoding RNAs that regulate EMT through targeting genes by translational silencing or mRNA degradation. Using bioinformatics software, we found that miR-30a could potentially interact with NFATc3. NFAT signaling is known to maintain podocyte function in areas such as protein synthesis [34], metabolism [35], cytoskeleton regulation [36] and degradation [37], and via regulation of the transcription of important genes for podocytes. Altered NFAT signaling can induce podocyte dysfunction, ultimately resulting in glomerulosclerosis [24] and the activation of fibronectin protein [10]. In the present study, we found that NFATc3 had higher expression than any other member of the NFAT family and was visibly enhanced after ADR treatment, suggesting that NFATc3 may play an important role in podocyte injury. Unequivocal evidence of whether or how miR-30a downregulates NFATc3 is still not available. However, this study shows that miR-30a downregulates NFATc3 by suppressing its nuclear translocation. The overexpression of miR-30a in podocytes enhances the prevention of cytoskeleton disorder or rearrangement of the actin cytoskeleton, a main factor of the complex architecture for podocyte function, suggesting that augmented miR-30a levels in podocytes inhibit the nuclear translocation of NFATc3 to protect the architecture and function of podocytes.

In summary, based on the observation that miR-30a is downregulated in podocyte injury models and glomeruli of FSGS patients, the present study demonstrates for the first time that miR-30a reduces EMT to protect podocyte function by targeting NFATc3. Thus, miR-30a might be a potential therapeutic target for inhibiting podocytopathy, and inhibition of it can be a potential diagnostic biomarker for podocyte injury.

## 4. Experimental Section

### 4.1. Human Samples

One sample was taken from a patient with FSGS at Department of Nephrology, the First Affiliated Hospital, Chongqing Medical University (Chongqing, China) and was identified by renal biopsy [25]. The control sample was taken from one patient with kidney rupture due to violence. Isolation of glomeruli from renal tissues by standard mechanical sieving technique was performed as described previously [38], snap-frozen in liquid nitrogen for RNA preparation, followed by cDNA synthesis and qPCR. Informed consent was acquired from all participants. This study was approved by the Chongqing Medical University Ethics Committee (Chongqing, China).

### 4.2. Animals and Treatment

All the experiments of animals in this study were approved by the Chongqing Medical University Animal Ethics Committee according to the “China Code of Practice for the Care and Use of Animals for Scientific Purposes”. BALB/c mice (6–7 weeks old) were used to generate adriamycin (ADR)-induced podocyte injury model [22,23]. All mice were purchased from the Animal Center Management, Chongqing Medical University, China. Three BALB/c mice per group were intravenously injected with Adriamycin (ADR, 10.5 mg/kg; Sigma, St. Louis, MO, USA). The control group was injected with an equivalent volume of normal saline (NS). At 4, 7, 11, 15 and 20 days post-injection, the mice were sacrificed and glomeruli were isolated by standard mechanical sieving technique as described before [38] and stored in liquid nitrogen for the next experiment.

### 4.3. Cell Culture and Transfection

Media and fetal bovine serum (FBS) were purchased from Invitrogen/ Gibco (Gibco, BRL Co., Ltd., Grand Island, NY, USA). HEK293T cells were cultivated in Dulbecco’s Modified Eagle Medium (DMEM) supplemented with 10% FBS, at 37 °C with 5% CO_2_. Conditionally immortalized mouse podocytes line 5 (MPC5) were cultured as described before [39] at 33 °C, 5% CO_2_ in RPMI 1640 medium supplemented with 10% FBS and recombinant mouse interferon gamma (10 U/mL; Peprotech, London, UK). MPC5 cells were maintained under nonpermissive conditions, at 37 °C in the absence of the recombinant mouse interferon gamma to induce differentiation, after at least 14 days, cells were treated with different concentrations of ADR at different time point depending on the next experimental setups.

For transfection, MPC5 and 293T cells were transfected with miR-30a mimics/inhibitor (50 nM) (RiboBio, Guangzhou, China) or pCDNA3.1(+)-Luc-NFATc3-3ʹUTR and pRL-CMV vectors by Lipofectamine 2000 reagent (Invitrogen, Grand Island, NY, USA).

### 4.4. Constructs and Luciferase Assays

Genomic DNA fragments containing 3ʹUTR of NFATc3 were obtained by PCR and inserted into pCDNA3.1-Luc reporter vector. Two miR-30a complementary sites with NFATc3-3ʹUTR were mutated with site directed mutagenesis. All the primer sequences were listed in Table 1. To normalize difference of transfection efficiency, pRL-CMV vector was applied and co-transfected for each well. 293T and MPC5 cells were seeded in 24-well plates and co-transfected with appropriate reporter and Renila plasmid and miR-30a mimics or the negative control of miRNA mimic (miR-NC) by Lipofectamine 2000 reagent. Thirty-six hours later, the dual-luciferase assay system (Promega, Madison, WI, USA) was used according to previous instructions [40].

**Table 1:** The sequences of the PCR primers for constructing NFATc3-3ʹUTR.

Name	Primer Sequences	Product Size
WT-NFATc3-3ʹUTR	Sense: TTTGCCCACCACGGACTG	2450 bp
	Antisense: TGAGGAGGAGCCTGGACTG	
Mut-NFATc3-3ʹUTR-BS1	Sense: GTTGAGAAGGCTTAATCTCCGTACATTTGCCCACAATGATTCTAT	-
	Antisense: ACAGAATCATTGTGGGCAAATGTACGGAGATTAAGCCTTCTCAAC	
Mut-NFATc3-3ʹUTR-BS2	Sense: AAGAAATTGGTTCTTGGTTAGTACATTTGCACTTGGGATTGTG	-
	Antisense: CACAATCCCAAGTGCAAATGTACTAACCAAGAACCAATTTCTT	

### 4.5. RNA Extraction and Real Time PCR Analysis

Total RNA in MPC5 cells and isolated glomeruli were extracted with TRIzol reagent (Ambion, Austin, TX, USA) following the manufacturer’s instructions. Quantitative real time PCR of miR-30a was amplified by internal reference primers (U6) and miR-30a specific primers (RiboBio, Guangzhou, China) with Revert Aid First Strand cDNA Synthesis kit (Fermentas, Burlington, ON, Canada) and SYBR Premix Ex Taq TM II (Takara, Dalian, China) according to the manufacturers’ protocols. The first-strand cDNA of protein-encoding genes was synthesized with random primers from Revert Aid First Strand cDNA Synthesis kit. miRNAs’ quantification were determined by Ultra SYBR Mixture (CWBIO, Beijing, China) with relevant primers of protein-encoding genes according to the manufacturers’ protocols. All the sequences of real time PCR primers were listed in Table 2. The comparative cycle threshold (*C*_t_) method was adopted to calculate the relative abundance of miRNA compared with U6 RNA. Levels of miR-30a and other mRNAs were respectively normalized to U6 RNA and 18s and calculated by using comparative cycle threshold (*C*_t_) method.

**Table 2:** The sequences of the real time PCR primers.

Gene	Gene Bank Association Number	Sense	Anti-Sense	Product Size
**NFATc1**	NM_198429	GCCTCGAACCCTATCGAGTG	AGTTATGGCCAGACAGCACC	121 bp
**NFATc2**	NM_010899	CGAGCTGGACTTTTCCATCCT	TCCAGGACATCATCCGGGTA	120 bp
**NFATc3**	NM_010901	ACGACGAGCTCGACTTCAAA	TGCAGCAGTCCATGATGTGG	181 bp
**NFATc4**	NM_023699	ACCTCCTGAGGGCTACAATG	CTCACTCACTTCCTCCAGGGT	145 bp
**NFATc5**	NM_018823	CAGCCAAAAGGGAACTGGAG	GAAAGCCTTGCTGTGTTCTG	173 bp
**Snail**	NM_011427	AGCCCAACTATAGCGAGCTG	CCAGGAGAGAGTCCCAGATG	150 bp
**Collagen I**	NM_007742	AGCACGTCTGGTTTGGAGAG	GACATTAGGCGCAGGAAGGT	112 bp
**Fibnectin**	NM_010233	CCCCAACTGGTTACCCTTCC	TGTCCGCCTAAAGCCATGTT	198 bp
**18s**	NR_003278	GTAACCCGTTGAACCCCATT	CCATCCAATCGGTAGTAGCG	151 bp

### 4.6. Protein Extraction and Western Blot Analysis

Cells were lysed with RIPA lysis buffer (Beyotime, Haimen, China). Protein concentration of each sample was measured by BCA reagent kit (Merck, Darmstadt, Germany). Cellular proteins (40 µg) was separated by SDS-PAGE gels electrophoresis, electro-transferred to PVDF membranes (Millipore Corporation, Billerica, MA, USA) and subsequently was blocked with 5% (*w*/*v*) no fat milk in TBST for 1 h at room temperature. The blots were probed with rabbit polyclonal antibody against desmin (Santa Cruz Biotechnology, Santa Cruz, CA, USA; 1:1000), nephrin (Abcam, Cambridge, MA, USA; 1:2000), E-cadherin (Santa Cruz Biotechnology; 1:1000), NFATc3 (Bioss, Beijing, China; 1:500) and mouse monoclonal anti-beta-tubulin (Santa Cruz Biotechnology; 1:3000, anti-beta-actin (Santa Cruz Biotechnology; 1:3000) overnight at 4 °C. The membranes were then incubated with goat Ig anti-rabbit IgG-HRP (Santa Cruz Biotechnology) and goat Ig anti-mouse IgG-HRP (Santa Cruz Biotechnology) for 1 h at room temperature the next day. Western Blot chemiluminescent HRP substrate Reagent (Millipore Corporation) was used to detect Western Blotting antibody. Normalization of proteic expression was used of internal control (β-tubulin or β-actin).

### 4.7. Immunofluorescence Staining and F-Actin Cytoskeleton Staining

After treatment by transfecting with miR-30a or miR-NC, cells growing on glass coverslips were fixed with pre-cold acetone for 15 min at −20 °C. Subsequently, cells were permeabilized and blocked respectively with 1% Triton X-100 (in PBS) and 5% BSA (in PBS) for 1 h at room temperature. For immunofluorescence staining, the rabbit polyclonal antibody against NFATc3 (Santa Cruz Biotechnology; 1:100) was used to probe NFATc3 overnight at 4 °C and goat anti-rabbit IgG-CFL 488 (Santa Cruz Biotechnology; 1:2000) was used to detect rabbit IgG for 1 h at room temperature. For F-actin cytoskeleton staining, cells were incubated in rhodamine-labeled phalloidin (Sigma; diluted in PBS containing 5% BSA, 1:1000) overnight at 4 °C.

Finally, nuclei were stained by DAPI (Santa Cruz Biotechnology; 1:5000) at room temperature for 5 min. All the coverslips were washed with PBS for three times and then softly rinsed with water as following. The coverslips were immobilized on the glass slides by 50% glycerol in PBS and viewed under a fluorescence microscope (ECLIPSE Ti-s, Nikon, Tokyo, Japan); and relevant images were taken with a SPOT Diagnostic (Sterling Heights, MI, USA) CCD camera.

### 4.8. Statistical Analysis

All the data were presented as the mean ± SD. Student’s *t*-test was employed for the statistical analysis of two independent groups by GraphPad Prism 5 software (GraphPad Software, Inc., La Jolla, CA, USA). For all tests, *p* < 0.05 (*) or *p* < 0.01 (**) was considered statistically significant. All experiments were performed at least three times.

## 5. Conclusions

In summary, based on the observation that miR-30a is downregulated in podocyte injury models and glomeruli of FSGS patients, the present study demonstrates for the first time that miR-30a inhibits EMT to protect podocyte functions by targeting NFATc3. Thus, miR-30a might be a potential therapeutic target for inhibiting podocytopathy, and its inhibition may be a potential diagnostic biomarker for podocyte injury.

## Abbreviations

MicroRNAs (miRNAs); microRNA-30a (miR-30a); Epithelial-to-mesenchymal transition (EMT); nuclear factor of activated T cells 3 (NFATc3); chronic kidney diseases (CKDs); focal segmental glomerulosclerosis (FSGS); diabetic nephropathy (DN); glomerular basement membrane (GBM); zonula occludens-1 (ZO-1); Transforming growth factor beta (TGFβ); nuclear factor of activated T cells (NFAT); 3’-untranslated region (3’UTR); non-small cell lung cancer (NSCLC); hepatocellular carcinoma (HCC); adriamycin (ADR).
